# Evaluating availability and price of essential medicines in Boston area (Massachusetts, USA) using WHO/HAI methodology

**DOI:** 10.1186/s40545-016-0059-5

**Published:** 2016-04-05

**Authors:** Abhishek Sharma, Lindsey Rorden, Margaret Ewen, Richard Laing

**Affiliations:** Department of Global Health, Boston University School of Public Health, Boston, MA USA; Center for Global Health and Development, Boston University School of Public Health, Boston, MA USA; Precision Health Economics, Boston, MA USA; Health Action International, Amsterdam, The Netherlands

## Abstract

**Background:**

Many patients even those with health insurance pay out-of-pocket for medicines. We investigated the availability and prices of essential medicines in the Boston area.

**Methods:**

Using the WHO/HAI methodology, availability and undiscounted price data for both originator brand (OB) and lowest price generic (LPG) equivalent versions of 25 essential medicines (14 prescription; 11 over-the-counter (OTC)) were obtained from 17 private pharmacies. The inclusion and prices of 26 essential medicines in seven pharmacy discount programs were also studied. The medicine prices were compared with international reference prices (IRPs).

**Results:**

In surveyed pharmacies, the OB medicines were less available as compared to the generics. The OB and LPG versions of OTC medicines were 21.33 and 11.53 times the IRP, respectively. The median prices of prescription medicines were higher, with OB and LPG versions at 158.14 and 38.03 times the IRP, respectively. In studied pharmacy discount programs, the price ratios of surveyed medicines varied from 4.4–13.9.

**Conclusions:**

While noting the WHO target that consumers should pay no more than four times the IRPs, medicine prices were considerably higher in the Boston area. The prices for medicines included in the pharmacy discount programs were closest to WHO’s target. Consumers should shop around, as medicine inclusion and prices vary across discount programs. In order for consumers to identify meaningful potential savings through comparison shopping, price transparency is needed.

**Electronic supplementary material:**

The online version of this article (doi:10.1186/s40545-016-0059-5) contains supplementary material, which is available to authorized users.

## Background

Lack of regular access to essential medicines remains a major public health concern globally. Essential medicines are identified by the World Health Organization (WHO) as those medicines which meet the global health needs of the majority population. The WHO Model Essential Medicines List is updated every two years in a transparent process [1]. While access has improved considerably since the introduction of the essential medicines concept in 1977, one-third of the world’s population is still not treated with the needed medicines [2–5]. Millar et al highlighted the potential value of using the WHO Model Essential Medicines List to reduce costs and provide more equitable access to low-income patients in the United States (US) [2].

In low- and middle-income countries (LMICs), as many as 90 % of the population pay out-of-pocket (OOP) for their medicines [3, 6]. The US has also seen a shift towards high-deductible insurance plans, within the last decade. The majority (78 %) of plans, covering medical procedures and prescription medicines, now have a general deductible (the amount that must be paid OOP before an insurer will pay any expenses), half of which are over $1,000 [7]. Low medicine availability, high prices and poor affordability are key barriers to medicine access [5, 8–12]. In many high-income countries including the US, there are growing concerns about reduced medicine access for reasons including high medicine prices and copayments/deductibles, uninsured populations, lack of transparency in medicine price components, and health agencies’ poor ability to negotiate procurement prices [13–17].

In 2001, a resolution (WHA 54.11) endorsed by the Member States of the World Health Assembly called for a standardized methodology to monitor medicines prices to help improve access [18]. In response, the World Health Organization/Health Action International (WHO/HAI) Project on Medicine Prices and Availability was established. The primary aim of this project was to develop a standardized method to measure medicines’ prices, availability, affordability and price components in a reproducible way so as to allow international comparisons over time. In 2003, after testing in nine countries, the standard WHO/HAI methodology was released, with a second edition published in 2008 [19]. To assess the surveyed medicines’ consumer prices, WHO/HAI methodology employs international reference prices (IRPs) as an external benchmark. To measure prices, a median price ratio (MPR) is calculated by comparing the median consumer price of a given medicine with the respective IRP. International reference prices used in this survey were taken from the 2013 Management Sciences for Health (MSH) International Drug Price Indicator Guide [20]. The MSH reference prices, first published in 1986, are procurement prices obtained from both sellers and buyers and collected from government agencies, pharmaceutical suppliers, and international development organizations. The MSH prices are widely accepted as an appropriate reference standard [9]. These MSH procurement prices report the actual prices obtained by non-profit suppliers and government tenders (see Additional file 1: List of price sources for 2013 MSH Drug Price Indicator Guide), the robust nature of this data ensures international comparability. Governments should be procuring medicines on the international market at close to IRPs. But patient prices in the private sector have to take into account additional costs in the pharmaceutical supply chain (mark-ups, tariffs, taxes and other costs). Because of these additional costs, WHO has set a target of four times the IRP for patient prices in the private sector. Recognizing medicine availability and prices as important components of access, the WHO medium term strategic plan 2008–2013 defines global and national targets for generic essential medicines, targeting 80 % availability in all sectors and median consumer prices to be no more than four times the IRP [21].

The WHO/HAI Project has been successful in developing a standard method for measuring price, availability, and affordability of essential medicines. As of 2014, more than 100 surveys had been conducted across the world, highlighting variations in medicine availability and prices by region, therapeutic category, and sector [9, 22]. The surveys provide transparency in price and availability reporting and inform medicines procurement globally.

In 2009, Cameron et al reported on medicine availability and prices in 36 developing and middle- income countries [9]. A key finding was that in the private sector the average MPR of originator brands (OB) by economic region varied from 13.8 to 40.9. For generic medicines, the average MPR by economic region varied from 9.8 to 11. The price ratios were adjusted for purchasing power parity.

In 2012, the US accounted for 35 % of the global spending on medicines [23]. Americans face a high burden of medicine expenditure owing to a combination of unregulated prices and high OOP expenditures: an average per-capita OOP spending of USD 758 on medicines in 2012 [24]. In 2014, an estimated 22 % of the patients did not fill a prescription or skipped prescription doses because of the cost [25]. Patients who are uninsured, elderly, low income, or with high insurance copays are disproportionately unlikely to fill their prescriptions [17]. Therefore, concerns about medicine access, especially due to high prices, persist in the US.

There is a widespread perception that generic medicines, which now represent 86 % of US prescriptions [7], are available at competitive prices. While generics are cheaper than the OB, the nature of generic price competition in the United States sometimes leads to aberrations such as unexpected price hikes. For instance, the price of daily average dose of albendazole, an older broad spectrum anti-parasitic medicine, rose from USD 5.92 in 2010 to USD 11.96 in 2013, while it is less than USD 1 in many countries [5].

Consumer Reports regularly investigates the availability and prices of a limited number of generic medicines across various pharmacy options available to consumers in the US [24, 26–28]. They also compare the OB and generic medicines across chain and independent pharmacies, but do not compare consumer prices to the IRP.

In the relative absence of surveys of the actual prices paid by consumers for prescription medicines in the US, we believe our initial survey in the Boston area can indicate whether the WHO/HAI methodology would be applicable, and allow comparisons with the studies undertaken by Consumer Reports. Medicines included in this survey are used globally, treat common conditions, and appear on most US treatment guidelines. Many of the surveyed medicines were included in the 2009 study of medicine availability and prices in 36 developing and middle-income countries [9].

Our Boston study investigated prices and availability of OB and generic essential medicines across chain, independent, big-box retail stores, and in-store supermarket pharmacies. Prices were obtained for both generic and OB products, and were then compared with the MSH IRPs. To our knowledge, there are no peer-reviewed studies assessing the availability and prices of OB and generic medicines in the US using the WHO/HAI methodology.

## Methods

A modified version of the WHO/HAI methodology was employed. A typical WHO/HAI survey collects data on availability and prices of a specified list of essential medicines plus supplementary medicines chosen by the investigator, and uses a standardized sampling frame including public-sector health facilities and registered private-sector retail pharmacies along with any other sector such as non-governmental or mission hospitals. Since the US lacks public sector distribution of medicines, we analyzed the availability and prices of 25 essential medicines in a representative sample of private-sector retail pharmacies (chain and independent) and 26 essential medicines offered by private-sector pharmacy and pharmacy discount programs in the Boston area. Data on some additional medicines were collected to provide a more robust sample of discount schemes. The WHO/HAI methodology also assesses the affordability of medicines, expressed as the number of day’s wages required by the lowest paid unskilled government worker to purchase a month’s supply of medicines for chronic conditions and a weeks’ supply for acute conditions. Accurately identifying the wage of a government worker in the Boston area is difficult hence affordability was not assessed.

### Sampling

#### Survey facilities

A list of currently licensed retail pharmacies (chain and independent) in zip codes within the cities of Boston, Cambridge, and Brookline was obtained from the Massachusetts Health Care Safety and Quality website [29]. Systematic random sampling was employed to select 10 chain pharmacies from this list. Each was then matched to an independent pharmacy in close proximity, resulting in a total survey sample of 20 pharmacies. This study also included a sample of seven pharmacy discount programs offered by big-box retail stores as well as in-store or freestanding pharmacies, including Walmart/Sam’s Club, Target, Hannaford, Walgreens, CVS, and Jewel-Osco. These programs offer a selection of medicines usually at prices of $4 per month or $10 for three months. These programs were selected using non-probability, convenience sampling and the price data were collected, using public online sources, for the surveyed medicines in these programs. See Additional file 1: Survey facilities random sampling (methods) for further details.

#### Survey medicines

For the facility survey, 14 prescription medicines were selected from the WHO/HAI global core medicines list, and 11 commonly-used over-the-counter (OTC) medicines. The survey basket for the pharmacy discount programs consisted of 26 medicines identified from WHO/HAI global and regional (Latin America and the Caribbean) core medicine lists [3, 10]. All medicines were strength and dosage-form specific. Table 1 lists the survey medicines. There were 14 medicines common to both pharmacy facility and discount program surveys. All surveyed medicines are commonly used and have an available IRP [9].

**Table 1:** List of medicines surveyed

Medicines	Strength	Dosage form/Unit	Pack size (recommended)^a^	Originator Brand
A. Over-the-counter medicines				
Acetaminophen/Paracetamol	325 mg	Tab/cap	100	Tylenol (McNeil)
Acetylsalicylic Acid	500 mg	Tab/cap	100	Asprin (Bayer)
Cimetidine	200 mg	Tab/cap	30	Tagamet (GSK)
Clotrimazole vaginal cream	1 %	Gram	24	Clotrimin (MSD)
Diphenhydramine HCl	25 mg	Tab/cap	100	Benadryl (McNeil)
Hydrocortisone topical cream	1 %	Gram	51	–
Ibuprofen	200 mg	Tab/cap	200	Advil (Pfizer)
Loratadine	10 mg	Tab/cap	30	Claritin (MSD)
Miconazole Nitrate topical cream	2 %	Gram	9	Monistat (McNeil)
Omeprazole	20 mg	Tab/cap	42	Prilosec (AstraZeneca)
Ranitidine	150 mg	Tab/cap	80	Zantac (Boehringer)
B. Prescription medicines				
Amitriptyline	25 mg	Tab/cap	100	Tryptizol (MSD)
Amoxicillin	500 mg	Tab/cap	21	Amoxil (GSK)
Atenolol	50 mg	Tab/cap	60	Tenormin (AstraZeneca)
Captopril	25 mg	Tab/cap	60	Capoten (BMS)
Ceftriaxone injection	1 g/vial	Vial	1	Rocephin (Roche)
Ciprofloxacin	500 mg	Tab/cap	10	Ciproxin (Bayer)
Co-trimoxazole suspension	8 + 40 mg/ml	Gram	100	Bactrim (Roche)
Diazepam	5 mg	Tab/cap	100	Valium (Roche)
Diclofenac	50 mg	Tab/cap	100	Voltarol (Novartis)
Glibenclamide	5 mg	Tab/cap	60	Daonil (Sanofi-Aventis)
Omeprazole	20 mg	Tab/cap	30	Losec (AstraZeneca)
Paracetamol (Acetaminophen)	24 mg/ml	Milliliter	60	Panadol (GSK)
Salbutamol inhaler	100 mcg/dose	Dose	200	Ventoline (GSK)
Simvastatin	20 mg	Tab/cap	30	Zocor (MSD)
C. Pharmacy discount program medicines				
Acute medicines				
Amoxicillin^b^	500 mg	Tab/cap	30	Amoxil (GSK)
Amoxicillin suspension	250 mg/5 ml	Milliliters	150 ml	Amoxil (GSK)
Azithromycin	500 mg	Tab/cap	3	Zithromax (Pfizer)
Ceftriaxone Injection^b^	1 g/vial	Vial	1	Rocephin (Roche)
Ciprofloxacin^b^	500 mg	Tab/cap	20	Ciproxin (Bayer)
Clotrimazole topical cream^b^	1 %	Gram	15 gram tube	Canesten (Bayer)
Diclofenac^b^	50 mg	Tab/cap	60	Voltarol (Novartis)
Furosemide	40 mg	Tab/cap	30	Lasix (Sanofi-Aventis)
Hydrochlorothiazide	25 mg	Tab/cap	30	Dichlotride (MSD)
Ibuprofen^b^	400 mg	Tab/cap	90	Brufen (Knoll)
Metronidazole	500 mg	Tab/cap	14	Flagyl (Sanofi-Aventis)
Omeprazole^b^	20 mg	Tab/cap	30	Prilosec (AstraZeneca)
Ranitidine^b^	150 mg	Tab/cap	60	Zantac (GSK)
Chronic medicines				
Amitriptyline^b^	25 mg	Tab/cap	90	Tryptizol (MSD)
Amlodipine	5 mg	Tab/cap	90	Norvasc (Pfizer)
Atenolol^b^	50 mg	Tab/cap	90	Tenormin (AstraZeneca)
Atorvastatin	10 mg	Tab/cap	90	Lipitor (Pfizer)
Captopril^b^	25 mg	Tab/cap	180	Capoten (BMS)
Clonazepam	2 mg	Tab/cap	90	Rivotril (Roche)
Diazepam^b^	5 mg	Tab/cap	90	Valium (Roche)
Enalapril	10 mg	Tab/cap	90	Renitec (MSD)
Fluoxetine	20 mg	Tab/cap	90	Prozac (Eli Lilly)
Glibenclamide^b^	5 mg	Tab/cap	90	Daonil (Sanofi-Aventis)
Metformin	850 mg	Tab/cap	180	Glucophage (BMS)
Phenytoin	50 mg	Tab/cap	90	Epanutin (Pfizer)
Simvastatin^b^	20 mg	Tab/cap	90	Zocor (MSD)

^a^For facility surveys (Table 1a-b), data collectors were instructed to obtain information for these medicine pack sizes (number of units). If not available, the information for the size immediately larger was collected

^b^Medicines common to both the pharmacy survey and pharmacy discount scheme surveys

### Data collection, entry, cleaning, and analysis

After prior notification, the data collectors (Master of Public Health (MPH) students undertaking practical fieldwork) visited the selected pharmacy facilities and identified themselves during October 2014. The data collectors obtained information, by physically inspecting the availability (in-stock) of the OB medicines and their generic equivalents on the day of survey. The data collectors also obtained information on the undiscounted retail prices of the OB and the lowest price generics (LPG) versions of the survey medicines using a standardized form developed by the WHO/HAI. These prices reflect the amount in US dollars that a patient without any health insurance or special medicine plan would pay to purchase a given medicine.

For the pharmacy discount programs, price data were obtained from public online sources during November 2014 (see Additional file 1: Pharmacy discount program analysis (methods)). The medicine unit prices collected from the facility survey were entered into the Excel-based WHO/HAI Medicine Prices Workbook, followed by double entry, automated and manual error-checking, and built-in automated analysis feature of the workbook [19]. The pharmacy discount program data were entered and analyzed using MS Excel. The facility survey workbooks have been submitted to HAI and will be posted on the HAI online price and availability database http://haiweb.org/what-we-do/price-availability-affordability/.

In the case of the facility survey, medicine availability is reported as the mean percentage of the retail pharmacies (overall and stratified by chain and independent) where a given medicine was found. To facilitate international comparisons, medicine-specific median price ratios (MPR) were calculated when prices were available from at least four facilities. The MPR refers to the ratio of a medicine’s local median unit price (across pharmacies) as compared to the 2013 MSH international median unit reference price [19, 20].

To summarize the MPRs of OB and LPG medicines, we performed ‘all medicines’ and ‘matched pair’ MPR analyses, overall and by pharmacy-type. While the ‘all medicines’ analysis considers all the available MPRs for each survey medicine, the ‘matched pair’ analysis considers the available MPRs for only those survey medicines which existed in OB-LPG pairs. Using statistical software SAS version 9.3, we conducted hypothesis testing to see if availability and prices varied among pharmacies at alpha significance of 0.05 (marginal significance if *p*-value between 0.05–0.06) (See Additional file 1: Statistical analysis: facility medicine availability and prices (results)).

For each of the pharmacy discount programs, medicine availability is reported as the percentage of the medicines included in pharmacy discount programs survey basket (See Table 1c) which were included in a given program. Furthermore, we calculated medicine-specific ‘price ratio’ using the following formula:

$$ Price\  Ratio = \frac{Discount\  program\  unit\  price\ (USD)}{MSH\  median\  unit\  international\  reference\  price\ (USD)} $$

## Results

The surveyed facilities varied in size, ranging from 720 to over 30,000 square feet. The space for pharmacy services including dispensing and products ranged in size from as much as 216 feet of shelf space for OTCs to no OTC shelving at all. Of the total 20 pharmacies sampled, data on OTC medicines were obtained from 17 pharmacies (10 chain; 7 independent). Only 14 pharmacies (8 chain; 6 independent) provided data on the prescription medicines. Pharmacy staff’s busy schedules or unwillingness to cooperate appeared to be the main reasons for the sample drop-outs in the case of prescription medicines. However, some independent pharmacies even refused to allow the collection of the OTC medicines information, which they referred to as “proprietary information”.

### Facility survey: availability of surveyed medicines

Table 2 summarizes the availability of OTC and prescription medicines, stratified by OB and generic equivalents, in chain and independent pharmacies. In general, the overall availability of OTC medicines was higher than the prescription medicines. The OB medicines were less available (prescription: 42.3 %; OTC: 73.8 %) as compared to the generic equivalents (prescription: 78.6 %; OTC: 85.6 %). However, this difference was statistically significant for prescription medicines only. The originator version of omeprazole was available in only 50 % of facilities; however the generic was available in 93 % of facilities. The OB version of OTC medicine clotrimazole, which was not available in any of the surveyed facilities, only had availability as generic in 58.8 % of facilities.

**Table 2:** Mean percentage availability of surveyed medicines in retail pharmacies

	Prescription medicines		Over-the-counter medicines	
	Originator Brand % (number of pharmacies)	Generic % (number of pharmacies)	Originator Brand % (number of pharmacies)	Generic % (number of pharmacies)
Chain	52.7 % (*n* = 8)	78.6 % (*n* = 8)	80.9 % (*n* = 10)	94.5 % (*n* = 10)
Independent	28.6 % (*n* = 6)	76.6 % (*n* = 6)	63.6 % (*n* = 7)	72.7 % (*n* = 7)
Overall	42.3 % (*n* = 14)	78.6 % (*n* = 14)	73.8 % (*n* = 17)	85.6 % (*n* = 17)

Overall mean availability of originator brand (OB) and generic equivalent (GE) versions of over-the counter (OTC) medicines is not statistically different (*p*-value = 0.24). However, the overall mean availability of OB and GE versions of prescription medicines is statistically different (*p*-value < 0.001). Availability of OB and GE versions of matched pairs was not statistically different (*p*-value > 0.05) from each other for neither OTC nor prescription medicines, in both chain and independent pharmacies. Mean availability of GE of OTC medicines was statistically different (*p*-value = 0.01) in chain and independent pharmacies. There was no statistically significant difference (*p*-value = 1.0) in mean availability of GE of prescription medicines among chain and independent pharmacies. The mean availability of OB version were statistically different among chain and independent pharmacies, in case of both OTC (*p*-value = 0.001) and prescription (*p*-value < 0.001) medicines

For prescription medicines, the availability of generic equivalents was similar (*p*-value = 1.00) among the chain (78.6 %) and independent pharmacies (76.6 %). However, the OB prescription medicines were relatively less available (*p*-value < 0.001) in independent pharmacies (28.6 %) as compared to the chain counterparts (52.7 %).

In the case of OTC medicines, both the OB and generic equivalent medicines were statistically (*p*-value = 0.01) more available in chain pharmacies (80.9 and 94.5 %, respectively) as compared to those in independent pharmacies (63.6 and 72.7 %, respectively). See Additional file 1: Tables S1–S4.

### Facility survey: price of surveyed medicines

#### Over-the-counter medicines

Table 3 summarizes the MPRs of the surveyed OTC medicines. Overall, across all medicines in the analysis (Table 3a), the OB and LPG versions of surveyed OTC medicines were priced 21.33 (range: 11.41–41.24) and 11.53 (2.68–29.42) times the IRPs, respectively. Cimetidine had the highest MPR among both the OB (MPR: 41.24) and LPG medicines (MPR: 29.42). Loratadine and clotrimazole had the lowest MPRs among the OB and LPG medicines, which were 11.41 and 2.68 respectively.

**Table 3:** Summary of median price ratios (MPR) of the surveyed over-the-counter medicines in retail pharmacies

3(a). All Medicines analysis						
	Overall		Chain Pharmacies		Independent Pharmacies	
	Originator Brand (medicines = 9)	Lowest Price Generic (medicines = 11)	Originator Brand (medicines = 9)	Lowest Price Generic (medicines = 11)	Originator Brand (medicines = 9)	Lowest Price Generic (medicines = 9)
Median MPR	21.33	11.53	20.81	11.53	17.98	9.43
Minimum, Maximum MPR	11.41, 41.24	2.68, 29.42	11.41, 143.13	2.68, 112.08	11.69, 37.27	2.55, 31.58
3(b). Matched pair analysis						
	Originator Brand	Lowest Price Generic	Originator Brand	Lowest Price Generic	Originator Brand	Lowest Price Generic
	OB-LPG pairs = 9		OB-LPG pairs = 9		OB-LPG pairs = 8	
Median MPR	21.33	14.56	20.81	15.81	17.81	9.50
Minimum, Maximum MPR	11.41, 41.24	5.40, 29.42	11.41, 143.13	5.79, 112.08	11.69, 35.15	3.55, 31.58

In 'all medicines' analysis, the overall median MPRs for the originator brand (OB) and lowest price generic (LPG) versions of the over-the-counter (OTC) medicines were marginally different (*p*-value = 0.056). In chain pharmacies, the median MPRs of the OB and LPG versions were not statistically different (*p*-value = 0.11). Whereas in the independent pharmacies, the median MPRs of the OB and LPG were statistically different (*p*-value = 0.004). Furthermore, the median MPRs of the OB version of OTC medicine in chain pharmacy were not statistically different (*p*-value = 0.96) from that in the independent pharmacy. Similarly, the median MPRs of the LPG versions were marginally different (*p*-value = 0.503) among the chain and independent pharmacies.

In 'matched pair' analysis, the overall median MPRs of the OB and LPG versions of OTC medicines were statistically different (*p*-value = 0.03). Within both chain and independent pharmacies, the median MPRs of the OB and LPG versions of OTC medicines were statistically different (chain *p*-value = 0.008; independent *p*-value = 0.03) from each other

The analysis of matched pairs (Table 3b) showed that overall the OB and LPG versions of the same products were priced 21.33 and 14.56 times the IRP respectively. The median price premium for OB versions was 46.5 % (range: 23.4–133.8 %) over the LPG price. In the case of both chain and independent pharmacies, the median MPRs of the OB were statistically higher (chain *p*-value = 0.008; independent *p*-value = 0.03) than that of the LPG medicines.

Across all medicines in the analysis, the unit price of OB medicines was 15.7 % higher in chain pharmacies than in independent pharmacies. The price of LPG medicines was 22.3 % higher in chain pharmacies than in independents. In the matched pairs analysis, the median MPRs of both OB and LPG versions of OTC medicines were higher in chain pharmacies as compared to independent pharmacies, however, the differences were not statistically significant (OB *p*-value = 0.81; LPG *p*-value = 0.20). Also see Additional file 1: Tables S5 and S7.

#### Prescription medicines

Across ‘all medicines’ (Table 4a), the OB and LPG versions of prescription medicines were 158.14 (range: 16.43–655.09) and 38.03 (12.52–155.46) times, respectively, the IRPs. Considering LPG versions, the median MPR in chain pharmacies (39.54) was higher than that in independent pharmacies (31.28), though this difference was not statistically significant (*p*-value = 0.31). In contrast, the median MPR for OB versions was higher in independent pharmacies (188.56) than in chain pharmacies (180.29), however no significant difference was found (*p*-value = 0.86). Notably, the MPR was calculated for only three prescription medicines pairs in independent pharmacies due to low availability.

**Table 4:** Summary of median price ratios (MPR) of the surveyed prescription medicines in retail pharmacies

4(a). All Medicines analysis						
	Overall		Chain Pharmacies		Independent Pharmacies	
	Originator Brand (medicines = 10)	Lowest Price Generic (medicines = 13)	Originator Brand (medicines = 8)	Lowest Price Generic (medicines = 12)	Originator Brand (medicines = 3)	Lowest Price Generic (medicines = 10)
Median MPR	158.14	38.03	180.29	39.54	188.56	31.28
Minimum, Maximum MPR	16.43, 655.09	12.52, 155.46	29.29, 663.30	19.15, 168.73	29.52, 655.09	5.37, 122.38
4(b). Matched pair analysis						
	Overall		Chain Pharmacies			
	Originator Brand	Lowest Price Generic	Originator Brand	Lowest Price Generic		
	OB-LPG pairs = 10		OB-LPG pairs = 8			
Median MPR	158.14	35.15	180.29	39.54		
Minimum, Maximum MPR	16.43, 655.09	12.52, 98.57	29.29, 663.30	28.73, 115.79		

In 'all medicines' analysis, overall median MPRs of originator brand (OB) and lowest price generic (LPG) versions of the prescription medicines were statistically different (*p*-value = 0.04). In chain pharmacies, the median MPRs of OB and LPG versions were statistically different (*p*-value < 0.05). Significance could not be calculated among independent pharmacies due to low availability of originator products.

In 'matched pair' analysis, too few pairs of sampled prescription medicines were available in independent pharmacies to make meaningful comparisons within such facilities. The overall median MPRs of the OB and LPG versions of prescription medicines were statistically different (*p*-value = 0.03), both overall and in case of chain pharmacies

In ‘matched pair’ analysis (Table 4b), the median MPRs of the OB and LPG versions of prescription medicines were statistically different from each other, both overall and within chain pharmacies. The median OB price premium was 299.1 % over the LPG price, ranging to as high as 1943.2 % in case of diazepam. Also see Additional file 1: Tables S6 and S8.

### Pharmacy discount programs

#### Inclusion

Table 5 summarizes medicine inclusion and prices in pharmacy discount programs. Among the seven pharmacy discount programs surveyed, the overall inclusion percentage of studied medicines was variable, ranging from 42.3 % (Walmart/Sam’s Club and Target $4/$10 program) to 100 % (Target prescription saver program). Overall, acute medicines were more frequently included than chronic medicines, with a mean inclusion of 71.4 % compared to 60.2 %, respectively. All pharmacy discount programs had higher acute medicine inclusion than chronic, except for the higher-priced Target prescription saver program, where 100 % of surveyed acute and chronic medicines were included.

**Table 5:** Inclusion and price ratio of studied medicines in the pharmacy discount program

Type of Medicine	Walmart/Sam’s Club		Target Prescription Saver		Target $4/$10		Hannaford		Walgreens		CVS		Jewel-Osco		Overall/Summary	
	Inclusion (%)^a^	Price Ratio [median (min, max)]^b^	Inclusion (%)^a^	Price Ratio [median (min, max)]^b^	Inclusion (%)^a^	Price Ratio [median (min, max)]^b^	Inclusion (%)^a^	Price Ratio [median (min, max)]^b^	Inclusion (%)^a^	Price Ratio [median (min, max)]^b^	Inclusion (%)^a^	Price Ratio [median (min, max)]^b^	Inclusion (%)^a^	Price Ratio [median (min, max)]^b^	Mean Inclusion (%)^a^	Median of Price Ratios [min, max]
Acute *n* = 12^c^	50.0 %	4.4 (3.3, 18.8)	100.0 %	16.1 (3.3, 70.7)	50.0 %	4.8 (3.3, 18.8)	100.0 %	16.3 (3.3, 74.3)	66.7 %	8.3 (4.4, 25.3)	58.3 %	13.3 (3.3, 46.7)	75.0 %	5.4 (3.5, 18.7)	71.4 %	8.3 (4.4, 16.3)
Chronic *n* = 14^c^	35.7 %	5.4 (3.3, 16.8)	100.0 %	15.0 (2.9, 81.3)	35.7 %	6.7 (3.3, 15.2)	92.9 %	5.4 (3.3, 70.1)	57.1 %	8.2 (2.5, 16.8)	50.0 %	6.5 (3.3, 20.2)	50.0 %	4.7 (3.3, 16.8)	60.2 %	5.0 (4.1, 13.7)
Overall Inclusion and Price Ratios [Median (min, max)] *n* = 26	42.3 %	4.4 (3.3, 18.8)	100.0 %	13.9 (2.9, 81.3)	42.3 %	4.4 (3.3, 18.8)	96.2 %	13.3 (1.4, 74.3)	61.5 %	8.2 (2.5, 25.3)	53.8 %	8.2 (3.3, 46.7)	61.5 %	5.3 (3.3, 18.7)		

^a^Inclusion refers to the percentage of total surveyed medicines offered by a given pharmacy discount program

^b^Compares median of the calculated price ratios to the MSH median unit reference price

^c^ *n* refers to the total number of medicines surveyed for acute and chronic medicines, respectively

#### Prices

Table 5 shows that among the surveyed pharmacy discount programs, the median of the MPR in which the discount program prices were compared to IRPs for the acute and chronic medicines were found to be 8.3 (range: 4.4–16.3) and 5.0 (4.1–13.7), respectively. Walmart/Sam’s Club and target $4/10 discount programs had the lowest overall MPR of the programs at 4.4 (range: 3.3–18.8), whereas Target Prescription saver had the highest MPR of the programs at 13.9 (range: 2.9–81.3). Also see Additional file 1: Table S9.

### Limitations of the study

Despite the strengths of the WHO/HAI methodology, there are some limitations of this study. First, we assessed the availability and prices for a specific list of medicines and did not account for other strengths, dosage forms or therapeutic alternatives. Due to the low availability of OB prescription products in independent pharmacies, we were unable to undertake meaningful ‘matched pair’ analysis of all prices. All pharmacists reported it would take them less than 24 hours to obtain specific prescription medicines that were not available in-store. In the case of the pharmacy discount programs, we assessed if a medicine was included in a given program but did not assess the physical availability for dispensing at the respective pharmacies. Also, our analysis is based on the data collected on the day of survey and may not indicate availability and prices over time. In addition, we did not account for any discounts or insurance, which vary by patient. Lastly, the results of the facility study may not represent medicine availability and prices in other US states, however we provide an initial reference point for future studies to be conducted in North America.

## Discussion

To our best knowledge, this is the first WHO/HAI study to assess the availability and prices of essential medicines in private-sector retail pharmacies in the US. The availability of medicines is often suboptimal around the globe, for medicines to treat both chronic and acute conditions [9]. An analysis of findings from price and availability surveys conducted in 36 developing and middle-income countries in 2009 found the average availability of generics was 64 % in the private sector. Availability was very low in some countries (e.g. Chad 14 %, Philippines 34 %, Shangdong province, China 35 %) but good in others (e.g. Syria 98 %, Chennai, India 92 %). Price premiums paid for OBs compared to generics ranged from 152 % in the private sector in upper-middle income countries to over 300 % in low-income countries [9].

In our study, while few patients pay full list prices, assessing the actual prices paid was not possible given that discounts are highly variable and cannot be standardized. While it is possible to assess individual level prices paid using prescription claims data, this data does not include the population of interest, such as patients without health insurance (~33 million i.e. 10.4 % of people in the US in 2014) [30] and/or those who pay OOP. Furthermore, as mentioned earlier, more people are opting for high deductible insurance plans; the number increased from 11.4 million in 2011 to 15.5 million in 2013. Notably 49 % of these people were age 40 and over, an age group with expectedly higher healthcare and prescription needs [31].

Results of our survey show that overall availability was similar to WHO’s target of 80 %, except for OB prescription medicines, which had lower availability, especially in independent pharmacies. This may be due to several factors, including independent pharmacies’ ability to procure OB prescription medicines quickly, low demand for such products, as well as the consumer and insurer trend towards purchasing generics whenever possible. Notably, availability was assessed in an environment with a highly developed supply chain, where pharmacies routinely request and can avail less commonly prescribed medications within several hours from other sources as needed. In such cases, availability is not an absolute barrier to access. However, it can become a barrier to access when considering that some patients may not return to retrieve their medicines due to the inconvenience of returning. Furthermore, availability estimates reflect the market, reflecting difference in availability of OB medicines among chain and independent pharmacies. Mail-order pharmacies were not included in this study, however, they will impact the overall availability of medicines for patients. Our results were consistent with the Consumer Reports findings which showed substantial price variations across pharmacies, and that savings were realized when patients purchased certain generic medicines at big box-stores such as Walmart and Target and pharmacy discount programs, paying a discounted retail price [26–28].

While noting the WHO target that consumers should pay no more than four times the IRPs, we observed that medicine prices were high in the Boston area compared to IRPs. The OB and generic versions of OTC medicine prices in Boston area were as high as 21.33 and 14.56 times the MSH IRPs, respectively. The prices of prescription medicines were particularly high, with OB and generic versions at 158.14 (range 16.43–655.09) and 38.03 (12.52–155.46) times the IRP, respectively. These patient prices in Boston for the prescription medicines were very high when compared to the prices paid for the same 14 medicines in the private sector of some other high-income countries. A medicine price survey in Bahrain, undertaken in 2013 using the WHO/HAI methodology, showed that patients were paying 34.78 and 13.85 times IRPs for OBs and LPG respectively. In 2011 in Tatarstan Province in Russia, patients were paying 13.05 and 4.12 times IRPs for OB and LPG respectively. In 2010 in a high-income Caribbean country, patients were paying 61.44 and 17.33 times IRPs for OB and LPG respectively [22]. While the data was not adjusted for purchasing power parity, it is clear that patient prices in Boston were substantially higher than in these three countries.

Interestingly, the OTC medicines were cheaper in independent pharmacies than in chain pharmacies. Although the OB prescription medicines were higher priced in independent pharmacies, LPGs were higher priced in the chain pharmacies. A contributing factor may be the recent increased cost to register a generic medicine in the US [32].

While the prices obtained in this survey may seem high in relation to the IRPs, most patients receive insurance assistance or discounts from their health payer for prescription medicines (but not OTC medicines). For many of the prescription medicines surveyed, pharmacy discount programs are available. With the wide range in insurance assistance and discounts across health insurance plans and by product, consumers generally do not know what they will be expected to pay for a medicine when dispensed at the pharmacy using insurance. This lack of transparency can be disadvantageous for consumers. For uninsured patients, while it would be desirable to fill prescriptions through pharmacy discount programs, we don’t know if they are directed to these programs. Consumer Reports suggests that this is not the case [26].

This survey has been conducted in an intensely medicalized and urban environment. It is not clear what the results would be in other settings. The Boston survey will be conducted annually using the same methods to evaluate trends in medicine availability and price over time. It would be of interest to have similar surveys repeated in other areas to compare results.

Prescribers in the US should encourage consumers to consider the pharmacy discount programs, which offer generic medicines at lower prices. Unlike the facilities, the prices of medicines in the pharmacy discount programs were much closer to WHO’s target of four times the IRPs. Our analysis shows that the cheapest medicines, when not using insurance, are from the discount programs offered in big-box retail stores and in-store and free standing pharmacies. However, inclusion of a medicine in a given pharmacy discount program and the price offered varied across programs, providing a reason for consumers to shop around.

If the policy in the US is not to regulate medicine prices, but rather rely on retail price competition, then a transparent system is needed that allows consumers to easily check prescription medicine prices at different pharmacies in order to identify potential savings. These benefits will need to be balanced against the cost of membership fees and the challenges of travel to sites with discounted prices. Consumers must be empowered to choose facilities and payment options most beneficial to them, whether that’s paying with insurance at a traditional pharmacy, or foregoing insurance co-payments and opting for discounted medicines through pharmacy discount programs or other options. However, such decisions cannot be made without transparent prices that allow for comparison. The current lack of transparency even extended to our survey where 6 pharmacies (4 independent and 2 chains) failed to provide full price information for the prescription medicines surveyed.

## Conclusion

The responsibility for ensuring price transparency rests primarily with policy-makers. Comprehensive policies are needed that are legally binding. Consumers (as well as healthcare providers and others) must be able to easily access regularly updated medicine price information in order to make informed decisions about the treatments.

### Ethical approval

This study did not require an ethical approval.

## Additional file

Additional file 1: Supplementary details on survey methods, data and statistical analyses. (PDF 874 kb)
