# Supplementary material for: Evaluating availability and price of essential medicines in Boston area (Massachusetts, USA) using WHO/HAI methodology
Source: J Pharm Policy Pract. 2016 Apr 5;9:12. doi: 10.1186/s40545-016-0059-5 (PMC4822245; doi:10.1186/s40545-016-0059-5)
Supplement: Additional file 1: — Supplementary details on survey methods, data and statistical analyses. (PDF 874 kb) [file 40545_2016_59_MOESM1_ESM.pdf]

Additional file 1

Table of contents

Additional details about methods

Page 2: Survey facilities random sampling (methods)

Pages 3-5: Pharmacy discount program analysis (methods)

Page 6: Statistical analysis: facility medicine availability and prices (methods)

Detailed information about medicine availability (facility survey)

Page 7: Table S1. Availability of surveyed over-the-counter medicines in retail pharmacies

Page 8: Table S2. Availability of surveyed prescription medicines in retail pharmacies

Page 9: Table S3: Availability of surveyed over-the-counter medicines in retail pharmacies: chain vs. independent

Page 10: Table S4: Availability of surveyed prescription medicines in retail pharmacies: chain vs. independent

Detailed information about medicine prices (facility survey)

Page 11: Table S5: Median price ratio of surveyed over-the-counter medicines in retail pharmacies

Page 12: Table S6: Median price ratio of surveyed prescription medicines in retail pharmacies

Page 13: Table S7: Median price ratio of surveyed over-the-counter medicines in retail pharmacies: chain vs. independent

Pages 14-15: Table S8: Median price ratio of surveyed prescription medicines in retail pharmacies: chain vs. independent

Detailed information about medicine prices (pharmacy discount program survey)

Pages 16-18. Table S9: Price ratios of surveyed medicines in the studied pharmacy discount programs

Statistical analyses and Data sources for MSH International Reference Prices

Pages 19-30: Statistical analysis: facility medicine availability and prices (results)

Page 31-36: List of price sources for the 2013 MSH Drug Price Indicator Guide

Survey facilities random sampling (methods)

The sample frame was constructed using the Massachusetts Health Care Safety and Quality License Verification Site. An initial list of pharmacies was compiled from this site, filtered by profession (pharmacy) and license type (retail drug store). It was further restricted to only current licensed pharmacies in the cities of Boston, Brookline, and Cambridge (zip codes: 02108-02111, 02113-02116, 02118, 02120, 02124-02129, 02135-02136, 02138-02141, 02146, 02215, and 02446). Licensed pharmacies include chain, independent, and hospital outpatient pharmacies. Hospital outpatient pharmacies were excluded, leaving a sample frame of all current and licensed pharmacies in the three cities of interest. Of the total number of current pharmacies in Massachusetts (n=1,146), 65 pharmacies are in the cities of Boston, Brookline, and Cambridge. This list was sorted by chain pharmacies (n=46), followed by independent pharmacies (n=19). The sample of interest was a total of 20 pharmacies, including 10 chain pharmacies and 10 independent pharmacies to ensure adequate representation. Sampling began by selecting 10 chain pharmacies. The sampling interval was calculated as the number of chain pharmacies in the sampling frame (n=46) divided by the sample of interest (n=10) for an interval of 4.6. A random number, 0.2874, was generated in excel and then multiplied by the sample interval (4.6), yielding a sample start of 1.32. This result was rounded up to the next integer, beginning sampling with pharmacy #2. Using this sampling interval, 10 chain pharmacies were selected for survey. Ten independent pharmacies were then matched to the selected chain pharmacies based on proximity (zip code followed by distance to walk). One pharmacy was excluded from analysis due to unavailability of data. The list of randomly selected pharmacies is available at request.

Pharmacy discount program analysis (methods)

Pharmacy discount program features

Pharmacy discount program benefits apply to specific generics at commonly prescribed dosages. Prices of listed generics range from $3.99 to $15 for up to 30-day supply and $9.99 to $30 for up to 90-day supply. An enrollment or annual membership fee is required to participate in the majority of pharmacy discount program. Annual membership fees range from $7 per family at Hannaford to $35 per family at Walgreens. No enrollment fee is required to participate in Walmart or Sam's Club Retail Prescription Program. Most program discounts cannot be combined with other promotions or insurance, do not count towards a customer's insurance deductible, and cannot be used to discount a customer's copay. Program discounts can be redeemed in conjunction with insurance at Walmart and Sam's Club.

Pharmacy discount program selection

Convenience sampling was used to select big-box store pharmacy discount for survey. Program were included if they had the following characteristics: existence of a generic prescription retail discount program, a pharmacy operating in the Greater Boston area, published or searchable price list of medicines included in the program, and availability of at least 11 of the 26 (>40%) medicines included in the survey. Greater Boston is defined as Boston-Cambridge-Newton, MA Metropolitan Statistical Area. This is further defined as Boston, MA Metropolitan Division (*Norfolk county, Plymouth country, Suffolk County)* and Cambridge-Newton-Framingham, MA Metropolitan Division (*Essex County, Middlesex County*).[1] Availability is defined as the existence of a survey medicine in the published medicine price list associated with each retail discount program at the time of survey. A total of 7 big-box stores or free-standing pharmacies with retail discount program were included for survey.

Medicine selection

Medicines surveyed in the pharmacy discount program were taken from model lists developed by Health Action International (HAI) in partnership with the World Health

Organization (WHO) in the Project on Medicine Prices and Availability. Surveyed medicines include 11 of the 14 medicines on the WHO/HAI Global core list, and 15 of the 16 medicines on the Latin America and Caribbean (AMRO) Regional core list. The four medicines excluded were not available in the listed form in any of the pharmacy discount programs. The four medicines excluded are as follows: co-trimoxazole suspension, paracetamol suspension, salbutamol inhaler, and beclometasone inhaler. The strengths of surveyed medicines were identical to those included in the WHO/HAI model lists, with the exception of phenytoin, where 100 mg tablets was unavailable and 50 mg tablets were surveyed instead. We used MSH Median Buyer Reference Price for Phenytoin 50 mg. Pack sizes of surveyed medicines differ from those included in the WHO/HAI model lists, as quantities included in pharmacy discount programs are based on the most common 30-day supply for acute medicines and the most common 90-day supply for chronic medicines. Of note, clotrimazole 1% topical cream was unavailable in the 20mg tube form, as the most common pack size found was 15gram tubes. This smaller package was surveyed across programs; however, the international reference price is based on 20g tube packages. Amoxicillin suspension 100 ml bottles were only available in 3 out of the 7 programs, but 150 ml bottles were available in all programs, and thus this bottle size was surveyed. Quantities included in the 30-day and 90-day supplies vary slightly across programs, adding complexity to unit price comparisons across programs. For instance, the majority of programs define 90-day supply of enalapril as 90 tabs; however, Walgreens defines it as 180 tabs, and 60 tabs for 30-days. International median reference prices are most often based on several tenders and various pack sizes, some of which vary from the pack size of the medicines we surveyed, usually consisting of larger quantities than those surveyed. It must be noted that the CVS Health Savings Pass program offers 90-day supply only, and unit prices in the analysis are calculated on this quantity. For acute medicines where 30-day supply is reported, unit prices are expected to be marginally lower in the CVS program than the programs that offer 30-day supply quantities.

Prices of eligible generic prescription medicines within pharmacy discount programs:

- CVS

  90-day supply for $11.99

  30-day supply of antibiotics & anti-infectives for $10.99
- Walgreens

  30-day-supply for $5 (tier 1), $10 (tier 2) or $15 (tier 3)

  90-day-supply for $10 (tier 1), $20 (tier 2) or $30 (tier 3)
- Hannaford

  30–day supply $4.00

  90–day supply $9.99

  * Some prices calculated on per-drug basis using online searchable database.
- Jewel-Osco

  30-day-supply for $3.99

  90-day supply for $9.99
- Walmart/Sam’s Club

  30-day-supply for $4

  90–day supply $10
- Target $4/$10 Generics

  30-day-supply for $4

  90-day supply for $10
- Target Prescription Saver- price calculated on per-drug basis using online searchable database.

Statistical analysis: facility medicine availability and prices (methods)

We tested a series of hypothesis to assess how the percentage availability (outcome) of originator and generic equivalents versions of over-the counter and prescription medicines statistically vary across and among facilities. Using Shapiro Wilk test and visual data inspection, we found that the data often did not follow normal distribution. Also the sample sizes were small. Therefore, non-parametric tests comparing medians constituted the choice over the parametric tests which compare means; non-parametric tests are more conservative as these do not assumes normal distribution of variables. However, the WHO/HAI methodology rightly suggests to summarize percentage availability in terms of 'means' because the medians can be insensitive to few, extreme outliers. Therefore, we performed both the non-parametric (Wilcoxon paired signed rank test) and parametric (paired t-test) tests to see if the median and mean percentage availability, respectively, are same among two groups.

Also in case of hypothesis tests regarding medicine prices, the sample sizes were small and the data did not follow a normal distribution. Therefore we used non-parametric tests to compare median MPRs (Median Price Ratios) among two given groups. For 'all medicines (non-paired) analyses, we performed two-sample Wilcoxon rank sum tests. And for 'matched pair' analyses, we performed paired Wilcoxon signed rank test. All the statistical tests were performed using statistical software SAS version 9.3 using alpha significance level of 0.05 (0.06 was considered as borderline significance).

Detailed information about medicine availability (facility survey)

Table S1: Availability of surveyed over-the-counter medicines in retail pharmacies		
**Medicines**	**Availability ***	
	**Originator Brand**	**Generic**
Acetaminophen/ Paracetamol 325 mg	94.1%	100.0%
Acetylsalicylic Acid 500 mg	82.4%	88.2%
Cimetidine 200 mg	82.4%	47.1%
Clotrimazole vaginal cream 1%	0.0%	58.8%
Diphenhydramine HCl 25 mg	82.4%	88.2%
Hydrocortisone topical cream 1%	11.8%	94.1%
Ibuprofen 200 mg	94.10%	100.0%
Loratadine 10 mg	88.2%	94.1%
Miconazole Nitrate topical cream 2%	88.2%	94.1%
Omeprazole 20 mg	94.1%	94.1%
Ranitidine 150 mg	94.1%	84.2%
**Mean**	**73.8%**	**85.6%**
**Median (IQR)**<br>**[min, max]**	**88.2% (82.4%-94.1%)**<br>**[0%, 94.1%]**	**94.1% (86.2% - 94.1%)**<br>**[47.1%, 100%]**
Note: * refers to the percentage of the total surveyed private retail pharmacies (n=17) where a given medicine was available for purchase.		

**Table S2: Availability of surveyed prescription medicines in retail pharmacies**		
**Medicines**	**Availability ***	
	**Originator Brand**	**Generic**
Amitriptyline 25 mg	7.1%	92.9%
Amoxicillin 500 mg	28.6%	92.9%
Atenolol 50 mg	78.6%	100.0%
Captopril 25 mg	35.7%	100.0%
Ceftriaxone inj 1g/vial	64.3%	78.6%
Ciprofloxacin 500 mg	50.0%	92.9%
Co-trimoxazole 8+40 mg/ml	28.6%	57.1%
Diazepam 5mg	85.7%	100.0%
Diclofenac 50 mg	7.1%	100.0%
Glibenclamide 5 mg	21.4%	42.9%
Omeprazole 20 mg	50.0%	92.9%
Paracetamol 24mg/ml	0.0%	7.1%
Salbutamol inhaler 100 mcg/dose	57.1%	50.0%
Simvastatin 20 mg	78.6%	92.9%
**Mean availability**	**42.3%**	**78.6%**
**Median (IQR) [min, max]**	**42.9% (23.2% – 62.5%) [0.0%, 85.7%]**	**92.9% (62.5% - 98.2%) [7.1, 100.0%]**
Note: * refers to the percentage of the total surveyed private retail pharmacies (n=14) where a given medicine was available for purchase.		

Table S3: Availability of surveyed over-the-counter medicines in retail pharmacies: chain vs. independent

**Medicines**	**Availability***			
	**Chain Pharmacy (n=10)**		**Independent Pharmacy (n=7)**	
	Originator Brand	Generic	Originator Brand	Generic
Acetaminophen/ Paracetamol 325 mg	100.0%	100.0%	85.7%	100.0%
Acetylsalicylic Acid 500 mg	90.0%	100.0%	71.4%	71.4%
Cimetidine 200 mg	100.0%	80.0%	57.1%	0.0%
Clotrimazole vaginal cream 1%	0.0%	70.0%	0.0%	42.9%
Diphenhydramine HCl 25 mg	90.0%	90.0%	71.4%	85.7%
Hydrocortisone topical cream 1%	10.0%	100.0%	14.3%	85.7%
Ibuprofen 200 mg	100.0%	100.0%	85.7%	100.0%
Loratadine 10 mg	100.0%	100.0%	71.4%	85.7%
Miconazole Nitrate topical cream 2%	100.0%	100.0%	71.4%	85.7%
Omeprazole 20 mg	100.0%	100.0%	85.7%	85.7%
Ranitidine 150 mg	100.0%	100.0%	85.7%	57.1%
**Mean availability**	**80.9%**	**94.5%**	**63.6%**	**72.7%**
**Median (IQR) [min, max]**	**100% (90%-100%) [0%, 100%]**	**100% (95% - 100%) [70%, 100%]**	**71.4% (64.3% - 85.7%) [0%, 85.7%]**	**85.7% (64.3%-85.7%) [0%, 100%]**

Note: * refers to the percentage of the total surveyed private retail pharmacies where a given medicine was available for purchase.

Table S4: Availability of surveyed prescription medicines in retail pharmacies: chain vs. independent

**Medicines**	**Availability***			
	**Chain Pharmacy (n=8)**		**Independent Pharmacy (n=6)**	
	Originator Brand	Generic	Originator Brand	Generic
Amitriptyline 25 mg	12.5%	87.5%	0.0%	100.0%
Amoxicillin 500 mg	37.5%	87.5%	16.7%	100.0%
Atenolol 50 mg	87.5%	100.0%	66.7%	100.0%
Captopril 25 mg	50.0%	100.0%	16.7%	100.0%
Ceftriaxone inj 1g/vial	87.5%	87.5%	33.3%	66.7%
Ciprofloxacin 500 mg	75.0%	87.5%	16.7%	100.0%
Co-trimoxazole 8+40 mg/ml	37.5%	62.5%	16.7%	50.0%
Diazepam 5mg	100.0%	100.0%	66.7%	100.0%
Diclofenac 50 mg	12.5%	100.0%	0.0%	100.0%
Glibenclamide 5 mg	25.0%	37.5%	16.7%	50.0%
Omeprazole 20 mg	62.5%	87.5%	33.3%	100.0%
Paracetamol 24mg/ml	0.0%	12.5%	0.0%	0.0%
Salbutamol inhaler 100 mcg/dose	50.0%	50.0%	66.7%	50.0%
Simvastatin 20 mg	100.0%	100.0%	50.0%	83.3%
**Mean**	52.7%	78.6%	28.6%	78.6%
**Median (IQR)**<br>**[min, max]**	**50.0% (28.1% - 84.4%)**<br>**[0%, 100%]**	**87.5% (68.8% - 100%)**<br>**[12.5%, 100%]**	**16.7% (16.7% - 45.8%)**<br>**[0%, 66.7%]**	**100% (54.2% - 100%)**<br>**[0%, 100%]**

Note: * refers to the percentage of the total surveyed private retail pharmacies where a given medicine was available for purchase.

Table S5: Median price ratio of surveyed over-the-counter medicines in retail pharmacies

Medicine	Unit	MSH Reference Unit Price 2013 (in USD)	Median Unit Price in USD (n=Number of prices)		Ratio of median prices of OB and LPG	OB price premium over the LPG price	Median Price Ratio (with regard to MSH reference prices)	
			Originator Brand (OB)	Lowest Price Generic (LPG)			Originator Brand	Lowest Price Generic
Acetaminophen/ Paracetamol 325 mg	Tab/cap	0.0048	0.1024 (n=16)	0.0699 (n=17)	1.46	46.5%	21.33	14.56
Acetylsalicylic Acid 500 mg	Tab/cap	0.0050	0.0699 (n=14)	0.0299 (n=15)	2.34	**133.8%**	13.98	5.98
Cimetidine 200 mg	Tab/cap	0.0105	0.4330 (n=14)	0.3090 (n=8)	1.40	40.1%	41.24	29.42
Clotrimazole vaginal cream 1%	Gram	0.1034	-- (n=0)	0.2776 (n=10)	--	--	--	2.68
Diphenhydramine HCl 25 mg	Tab/cap	0.0074	0.1948 (n=14)	0.1099 (n=15)	1.77	77.3%	26.32	14.85
Hydrocortisone topical cream 1%	Gram	0.0409	0.1695 (n=2)*	0.1338 (n=17)	*	*	#	3.27
Ibuprofen 200 mg	Tab/cap	0.0072	0.0840 (n=16)	0.0389 (n=17)	2.16	115.9%	11.67	5.40
Loratadine 10 mg	Tab/cap	0.0759	0.8663 (n=15)	0.5143 (n=16)	1.68	68.4%	11.41	6.78
Miconazole Nitrate topical cream 2%	Gram	0.0161	0.3893 (n=15)	0.3091 (n=16)	1.26	25.9%	24.18	19.20
Omeprazole 20 mg	Tab/cap	0.0213	0.6902 (n=16)	0.5592 (n=16)	1.23	23.4%	32.40	26.26
Ranitidine 150 mg	Tab/cap	0.0260	0.4171 (n=16)	0.2998 (n=14)	1.39	39.1%	16.04	11.53
**Median (IQR)**					**1.46 (1.39-1.77)**	**46.5% (39.1% - 77.3%) [min, max = 23.4%; 133.8%]**	**21.33 ** (13.98-26.32)**	**11.53 ** (5.69-17.03)**

Notes: * Less than 4 prices were available to calculate median unit price for the originator hydrocortisone topical cream 1%. Therefore ratio of median prices of OB to LPG or the OB price premium were not calculated. # The median price ratios with reference to the MSH reference prices were not calculated if the respective number of unit prices available is less than 4. ** The calculation includes on only those medicines (n=9) for which MPR is available for both OB and LPG. Ratio of median prices of OB and LPG being ‘x’ means that the OB version was ‘x’ times the prices of the LPG version of a given medicine.

Table S6: Median price ratio of surveyed prescription medicines in retail pharmacies

Medicine	Unit	MSH Reference Unit Price 2013 (in USD)	Median Unit Price in USD (n=Number of prices)		Ratio of median prices of OB and LPG	OB price premium over the LPG price	Median Price Ratio (with regard to MSH reference prices)	
			Originator Brand (OB)	Lowest Price Generic (LPG)			Originator Brand	Lowest Price Generic
Amitriptyline 25 mg	Tab/cap	0.008	0.6609 (n=1)*	0.2599 (n=13)	2.54	#	#	32.49
Amoxicillin 500 mg	Tab/cap	0.0313	0.5144 (n=4)	0.3920 (n=13)	1.31	31.2%	16.43	12.52
Atenolol 50 mg	Tab/cap	0.0118	2.0165 (n=11)	0.3366 (n=14)	5.99	499.1%	170.89	28.53
Captopril 25 mg	Tab/cap	0.0144	1.9265 (n=5)	0.9415 (n=14)	2.05	104.6%	133.78	65.38
Ceftriaxone inj 1g/vial	Injection Vial	0.5887	85.59 (n=9)	22.39 (n=11)	3.82	282.3%	145.39	38.03
Ciprofloxacin 500 mg	Tab/cap	0.0418	7.9290 (n=7)	1.3490 (n=13)	5.88	487.8%	189.69	32.27
Co-trimoxazole 8+40 mg/ml	Suspension	0.0051	0.1869 (n=4)	0.3399 (n=8)	0.55	-45.0%	36.65	66.65
Diazepam 5mg	Tab/cap	0.008	5.2407 (n=12)	0.2565 (n=14)	20.43	1943.2%	655.09	32.06
Diclofenac 50 mg	Tab/cap	0.0064	6.3299 (n=1) *	0.9950 (n=14)	6.36	#	#	155.46
Glibenclamide 5 mg	Tab/cap	0.0067	2.5198 (n=3)	0.5167 (n=6)	4.88	#	#	77.12
Omeprazole 20 mg	Tab/cap	0.0213	8.7330 (n=7)	2.0996 (n=13)	4.16	315.9%	410.00	98.57
Paracetamol 24mg/ml	Milliliter	0.0069	-- (n=0)	0.2332 (n=1) *	--	#	#	--
Salbutamol inhaler 100 mcg/dose	Dose	0.0099	0.2923 (n=8)	0.2950 (n=7)	0.99	-0.9%	29.52	29.80
Simvastatin 20 mg	Cap/tab	0.0235	8.3663 (n=11)	1.2360 (n=13)	6.77	576.9%	356.01	52.60
**Median (IQR)**					**4.16 (2.05 – 5.99)**	**299.1% (49.6% - 67.9%) [min, max = -45.0% - 1943.2%]**	**158.14 ** (60.93 - 314.43)**	**38.03 ** (32.06 – 66.65)**

Notes: * Not a median value, but the exact price or price ratio, as only the product was found only in one pharmacy. # The median price ratios with reference to the MSH reference prices or the OB price premiums are not calculated if the respective number of prices available is less than 4. ** The calculation includes on only those medicines (n=10) for which MPR is available for both OB and LPG. Ratio of median prices of OB and LPG being 'x' means that the OB version was 'x' times the prices of the LPG version of a given medicine.

Table S7: Median price ratio of surveyed over-the-counter medicines in retail pharmacies: chain vs. independent

Medicine	Unit	MSH Reference Unit Price 2013 (USD)	Type	Median Unit Price in USD (n=Number of prices)		Ratio of median prices of Chain and independent pharmacy	Median Price Ratio (with regard to MSH reference prices)	
				Chain Pharmacy	Independent Pharmacy		Chain Pharmacy	Independent Pharmacy
Acetaminophen/Paracetemol 500 mg	Tab/cap	0.0048	Originator Brand	0.0999 (n=10)	0.13015 (n=6)	0.77	20.81	27.11
			Lowest Price Generic	0.0759 (n=10)	0.0459 (n=7)	1.65	15.81	9.56
Acetylsalicyclic Acid 500 mg	Tab/cap	0.0050	Originator Brand	0.0699 (n=9)	0.0899 (n=5)	0.78	13.98	17.98
			Lowest Price Generic	0.0290 (n=10)	0.0409 (n=5)	0.71	5.79	8.18
Cimeditine 200 mg	Tab/cap	0.0105	Originator Brand	0.4546 (n=10)	0.39135 (n=4)	1.16	43.30	37.27
			Lowest Price Generic	0.3090 (n=8)	-- (n=0)	--	29.42	#
Clotrimazole vaginal cream 1%	Gram	0.1034	Originator Brand	-- (n=0)	-- (n=0)	--	#	#
			Lowest Price Generic	0.2776 (n=7)	0.2442 (n=3)	1.14	2.68	#
Diphenhydramine HCI 25 mg	Tab/cap	0.0074	Originator Brand	0.2038 (n=9)	0.1789 (n=5)	1.14	27.54	24.18
			Lowest Price Generic	0.1249 (n=9)	0.07545 (n=6)	1.66	16.88	10.20
Hydrocortisone topical cream 1%	Gram	0.0409	Originator Brand	0.1784 (n=1)*	0.1605 (n=1)*	1.11*	#	#
			Lowest Price Generic	0.1427 (n=10)	0.10435 (n=6)	1.37	3.49	2.55
Ibuprofen 200 mg	Tab/cap	0.0072	Originator Brand	0.0840 (n=10)	0.08415 (n=6)	1.00	11.67	11.69
			Lowest Price Generic	0.0419 (n=10)	0.027 (n=7)	1.55	5.82	3.75
Loratadine 10 mg	Tab/cap	0.0759	Originator Brand	0.8663 (n=10)	0.9997 (n=5)	0.87	11.41	13.17
			Lowest Price Generic	0.5914 (n=10)	0.2695 (n=6)	2.19	7.79	3.55
Miconazole Nitrate topical cream 1%	Gram	0.0161	Originator Brand	2.3045 (n=10)	0.2776 (n=5)	8.30	143.13	17.24
			Lowest Price Generic	1.8045 (n=10)	0.193 (n=6)	9.35	112.08	11.99
Omeprazole 20 mg	Tab/cap	0.0213	Originator Brand	0.6902 (n=10)	0.74865 (n=6)	0.92	32.40	35.15
			Lowest Price Generic	0.5593 (n=10)	0.6726 (n=6)	0.83	26.26	31.58
Ranitidine 150 mg	Tab/cap	0.0260	Originator Brand	0.3845 (n=10)	0.45885 (n=6)	0.84	14.79	17.65
			Lowest Price Generic	0.2998 (n=10)	0.2452 (n=4)	1.22	11.53	9.43
**Median (IQR)****			**Originator Brand**			**0.96 (0.85-1.13)**	**20.81 (13.98 - 32.40)**	**17.98 (17.24-27.11)**
			**Lowest Price Generic**			**1.46 (1.16-1.66)**	**11.53 (5.80 - 21.57)**	**9.43 (3.75 - 10.20)**

Notes: * Not a median value, but the exact price or price ratio, as product only found in one pharmacy. # The median price ratios with reference to the MSH reference prices are not calculated if the respective number of prices available is less than 4. ** Calculated for all the medicines. Missing values excluded.

Table S8: Median price ratio of surveyed prescription medicines in retail pharmacies: chain vs. independent

Medicine	Unit	MSH Reference Unit Price 2013 (USD)	Type	Median Unit Price in USD (n=Number of prices)		Ratio of median prices of Chain and independent pharmacy	Median Price Ratio (with regard to MSH reference prices)	
				Chain Pharmacy	Independent Pharmacy		Chain Pharmacy	Independent Pharmacy
Amitriptyline 25 mg	Tab/cap	0.0080	Originator Brand	0.6609 (n=1) *	-- (n=0)	--	#	#
			Lowest Price Generic	0.2489 (n=7)	0.3147 (n=6)	0.79	31.11	39.33
Amoxicillin 500 mg	Tab/cap	0.0313	Originator Brand	0.4920 (n=3)	0.5367 (n=1) *	0.92	#	#
			Lowest Price Generic	0.5995 (n=7)	0.1680 (n=6)	3.57	19.15	5.37
Atenolol 50 mg	Tab/cap	0.0118	Originator Brand	2.0165 (n=7)	2.2250 (n=4)	0.91	170.89	188.56
			Lowest Price Generic	0.3390 (n=8)	0.3154 (n=6)	1.07	28.73	26.73
Captopril 25 mg	Tab/cap	0.0144	Originator Brand	1.9832 (n=4)	1.2837 (n=1) *	1.54	137.72	#
			Lowest Price Generic	0.9415 (n=8)	0.9785 (n=6)	0.96	65.38	67.95
Ceftriaxone injection 1g/vial	Injection Vial	0.5887	Originator Brand	85.5900 (n=7)	89.7150 (n=2)	0.95	145.39	#
			Lowest Price Generic	19.9900 (n=7)	31.7000 (n=4)	0.63	33.96	53.85
Ciprofloxacin 500 mg	Tab/cap	0.0418	Originator Brand	7.9290 (n=6)	8.5350 (n=1) *	0.93	189.69	#
			Lowest Price Generic	1.7490 (n=7)	0.4238 (n=6)	4.13	41.84	10.14
Co-trimoxazole 8+40 mg/ml	Suspension	0.0051	Originator Brand	0.18690 (n=3)	0.1511 (n=1) *	1.24	#	#
			Lowest Price Generic	0.3399 (n=5)	0.2080 (n=3)	1.63	66.65	#
Diazepam 5mg	Tab/cap	0.0080	Originator Brand	5.3064 (n=8)	5.2407 (n=4)	1.0	663.30	655.09
			Lowest Price Generic	0.2979 (n=8)	0.2065 (n=6)	1.44	37.24	25.81
Diclofenac 50 mg	Tab/cap	0.0064	Originator Brand	6.3299 (n=1) *	-- (n=0)	--	#	#
			Lowest Price Generic	1.0799 (n=8)	0.7832 (n=6)	1.38	168.73	122.38
Glibenclamide 5 mg	Tab/cap	0.0067	Originator Brand	2.5198 (n=2)	2.4750 (n=1) *	1.02	#	#
			Lowest Price Generic	0.5565 (n=3)	0.3400 (n=3)	1.64	#	#
Omeprazole 20 mg	Tab/cap	0.0213	Originator Brand	8.7330 (n=5)	9.4959 (n=2)	0.92	410.00	#
			Lowest Price Generic	2.4663 (n=7)	0.6684 (n=6)	3.69	115.79	31.38

Paracetamol 24mg/ml	Milliliter	0.0069	Originator Brand	-- (n=0)	-- (n=0)	--	#	#
			Lowest Price Generic	0.2332 (n=1) *	-- (n=0)	--	#	#
Salbutamol inhaler 100 mcg/dose	dose	0.0099	Originator Brand	0.2900 (n=4)	0.2923 (n=4)	0.99	29.29	29.52
			Lowest Price Generic	0.2950 (n=4)	0.2895 (n=3)	1.02	29.80	#
Simvastatin 20 mg	Cap/tab	0.0235	Originator Brand	8.3663 (n=8)	8.3897 (n=3)	1.00	356.01	#
			Lowest Price Generic	1.2362 (n=8)	0.7330 (n=5)	1.69	52.60	31.19
**Median (IQR)****			Originator Brand			**0.99 (0.93-1.02)**	**180.29 (143.47-369.51)**	**188.56 (109.04-421.82)**
			Lowest Price Generic			**1.63 (1.07-2.05)**	**39.54 (30.78 - 65.70)**	**31.28 (26.04-50.22)**
Notes: * Not a median value, but the exact price or price ratio, as only the product was found only in one pharmacy. # The median price ratios with reference to the MSH reference prices are not calculated if the respective number of prices available is less than 4. ** Calculated for all the medicines. Missing values excluded.								

Table S9. Price ratios of surveyed medicines in the studied pharmacy discount programs

**Medicine Name**	**Pack Size**	**MSH Median Buyer Reference Price in USD**	**Walmart/ Sam's Club**		**Target Rx Saver**		**Target $4/$10**		**Hannaford**		**Walgreens**		**CVS**		**Jewel-Osco**		**Median of Price Ratios by Medicine [Min, Max] n = #**
			Unit Price in USD	Price Ratio	Unit Price in USD	Price Ratio	Unit Price in USD	Price Ratio	Unit Price in USD	Price Ratio	Unit Price in USD	Price Ratio	Unit Price in USD	Price Ratio	Unit Price in USD	Price Ratio	
GLOBAL CORE MEDICINES																	
Amitriptyline 25 mg tab	90	0.0204	0.1111	5.4	0.1111	5.4	--	--	0.35	17.2	0.1111	5.4	0.1332	6.5	--	--	5.4 [5.4,17.2] n=4
Amoxicillin 500 mg tab	30	0.0305	0.1333	4.4	0.1333	4.4	0.1333	4.4	0.1333	4.4	0.1667	5.5	0.3663	12.0	0.1330	4.4	4.4 [4.4,12.0] n=7
Atenolol 50 mg tab	90	0.0066	0.1111	16.8	0.1333	20.2	0.1000	15.2	0.1333	20.2	0.1111	16.8	0.1332	20.2	0.1111	16.8	16.8 [15.2,20.2] n=7
Captopril 25 mg tab	180	0.0164	--	--	0.2367	14.4	--	--	1.15	70.1	--	--	0.0667	4.1	--	--	**
Ceftriaxone Injection 1g vial	1	0.4838	--	--	5.9900	12.4	--	--	35.95	74.3	--	--	--	--	--	--	**
Ciprofloxacin 500 mg tab	20	0.0380	0.1666	4.4	0.1667	4.4	0.2000	5.3	0.1667	4.4	0.1667	4.4	0.5995	15.8	0.1665	4.4	4.4 [4.4,15.8] n=7
Diazepam 5 mg tab	90	0.0043	--	--	0.3497	81.3	--	--	0.113	26.3	--	--	--	--	--	--	**
Diclofenac 50mg tab	60	0.0099	--	--	0.5752	58.1	--	--	0.5368	54.2	0.2500	25.3	--	--	0.0665	6.7	39.7 [6.7,58.1] n=4
Glibenclamide 5mg tab	90	0.0052	--	--	0.1111	21.4	--	--	--	--	--	--	--	--	--	--	**

Omeprazole 20 mg tab	30	0.0248	--	--	0.7400	29.8	--	--	0.345	13.9	0.5000	20.2	--	--	0.1330	5.4	17.0 [5.4,29.8] n=4
Simvastatin 20 mg tab	90	0.0238	--	--	0.5264	22.1	--	--	0.111	4.7	0.2222	9.3	--	--	0.1110	4.7	7.0 [4.7,22.1] n=4
REGIONAL MEDICINES																	
Amlodipine Besylate 5mg tab	90	0.0272	--	--	0.4733	17.4	--	--	0.1110	4.1	0.2220	8.2	--	--	0.1110	4.1	6.1 [4.1,17.4] n=4
Amoxicillin suspension 250mg/5ml	150 ml	0.0075	0.0267	3.6	0.0267	3.6	0.0267	3.6	0.0267	3.6	0.0333	4.4	0.0733	9.8	0.0266	3.5	3.6 [3.5,9.8] n=7
Atorvastatin 10mg tab	90	0.0312	--	--	1.0000	32.1	--	--	0.1110	3.6	--	--	--	--	--	--	**
Azithromycin 500mg tab	3	0.2510	--	--	7.0567	28.1	--	--	8.1370	32.4	--	--	--	--	1.3300	5.3	**
Clonazepam 2mg tab	90	0.0450	--	--	0.6963	15.5	--	--	0.2417	5.4	--	--	--	--	--	--	**
Clotrimazole topical cream 1%	15g tube	0.0158	--	--	1.0670	67.5	--	--	1.0630	67.3	--	--	--	--	--	--	**
Enalapril 10mg tab	90	0.0106	0.1111	10.5	0.1111	10.5	0.1111	10.5	0.5600	52.8	0.1111	10.5	0.1332	12.6	0.1110	10.5	10.5 [10.5,52.8] n=7
Fluoxetine 20mg tab	90	0.0199	--	--	0.1111	5.6	0.1333	6.7	0.1110	5.6	0.1111	5.6	0.1332	6.7	0.1110	5.6	5.6 [5.6,6.7] n=6
Furosemide 40mg tab	30	0.0100	0.1333	13.3	0.1333	13.3	0.1333	13.3	0.1333	13.3	0.0833	8.3	0.1332	13.3	0.1330	13.3	13.3 [8.3,13.3] n=7
Hydrochlorothiazide 25mg tab	30	0.0071	0.1333	18.8	0.1333	18.8	0.1333	18.8	0.1333	18.8	0.1667	23.5	0.1332	18.8	0.1330	18.7	18.8 [18.7,23.5]

																	n=7
Ibuprofen 400mg tab	90	0.0135	0.0444	3.3	0.0444	3.3	0.0444	3.3	0.0444	3.3	0.1111	8.2	0.0444	3.3	0.0443	3.3	3.3 [3.3,8.2] n=7
Metformin 850 mg tab	180	0.0147	0.0555	3.8	0.0555	3.8	0.0555	3.8	0.0556	3.8	0.0370	2.5	0.0666	4.5	0.0555	3.8	3.8 [2.5,4.5] n=7
Metronidazole 500mg tab	14	0.0168	--	--	0.5107	30.4	--	--	0.7686	45.8	--	--	0.7850	46.7	0.2850	17.0	38.1 [17,46.7] n=4
Phenytoin 50mg tab	90	0.1590^	--	--	0.4607	2.9	--	--	0.5264	3.3	--	--		--	--	--	**
Ranitidine 150 mg tab	60	0.0202	0.0667	3.3	0.0667	3.3	0.0667	3.3	0.0667	3.3	0.1667	8.3	0.0666	3.3	--	--	3.3 [3.3,8.3] n=6
**Median of Price Ratios by Pharmacy Discount Program [Min, Max]**				**4.4 [3.3, 18.8] n=11**		**15.0 [2.9, 81.3] n=26**		**5.3 [3.3, 18.8] n=11**		**13.3 [3.3, 74.3] n=25**		**8.2 [2.5, 25.3] n=16**		**10.9 [3.3, 46.7] n=14**		**5.3 [3.3, 18.7] n=16**	**8.2 [8.2,15.0]**

--medicine not available through program
 # n refers to the total number of survey medicines available at each individual program
^ international reference price is single unit price, not a median. Unit price based on single source, with larger pack size of 1,000 tablets.
 * prices may be higher in the following states: CA, HI, MN, MT, PA, TN, and WI
**Median of price ratio not included due to inclusion of medicine in less than four pharmacy discount programs

Statistical analysis: facility medicine availability and prices (results)

Results: Medicine Availability

Test S1:

Null Hypothesis: Availability of originator brand and generic versions of OTC medicines is same.

Alternate Hypothesis: Availability of originator brand and generic versions of OTC medicines is not same.

Alpha Significance level: 0.05

Statistical tests:

Parametric Paired t-test (comparing mean availability): p-value = 0.2445

Non-parametric Wilcoxon (paired) signed rank test (comparing median availability): p value = 0.0781.

Conclusion: Since the p-value was found to be higher than 0.05 in both tests, we do not have enough statistical evidence ($\alpha = 0.05$) to reject the null. Therefore, the availability of originator brand and generic versions of OTC medicines is same.

Test S2:

Null Hypothesis: Availability of originator brand and generic versions of prescription medicines is same.

Alternate Hypothesis: Availability of originator brand and generic versions of prescription medicines is not same.

Alpha Significance level: 0.05

Statistical tests:

Parametric Paired t-test (comparing mean availability): p-value = 0.0006. Reject the Null

Non-parametric Wilcoxon (paired) signed rank test (comparing median availability): p value = 0.0007.

Conclusion: Since the p-value was found to be less than 0.05 in both tests, we do have enough statistical evidence ($\alpha = 0.05$) to reject the null. Therefore, the availability of originator brand and generic versions of prescription medicines is not same.

Test S3:

Null Hypothesis (1): Availability of originator brand and generic versions of OTC medicines is same in chain pharmacies.

Alternate hypothesis: Availability of originator brand and generic versions of OTC medicines is not same in chain pharmacies.

Alpha Significance level: 0.05

Statistical tests:

Parametric paired t-test (comparing mean availability): p-value = 0.2111.

Non-parametric Wilcoxon (paired) signed rank test (comparing median availability): p-value= 0.3750.

Conclusion: Since the p-value was found to be higher than 0.05 in both tests, we do not have enough statistical evidence ($\alpha = 0.05$) to reject the null. Therefore, the availability of originator brand and generic versions of OTC medicines is same in chain pharmacies.

Test S4:

Null hypothesis (2): Availability of originator brand and generic versions of OTC medicines is same in independent pharmacies.

Alternate hypothesis: Availability of originator brand and generic versions of OTC medicines is not same in independent pharmacies.

Statistical tests:

Parametric paired t-test (comparing mean availability): p-value = 0.3869.

Non-parametric Wilcoxon (paired) signed rank test (comparing median availability): p-value= 0.1406.

Conclusion: Since the p-value was found to be higher than 0.05 in both tests, we do not have enough statistical evidence ($\alpha = 0.05$) to reject the null. Therefore, the availability of originator brand and generic versions of OTC medicines is same in chain pharmacies.

Test S5:

Null Hypothesis (3): Availability of originator brand version of OTC medicines is same in chain and independent pharmacies.

Alternate hypothesis: Availability of originator brand version of OTC medicines is same in chain and independent pharmacies.

Alpha Significance level: 0.05

Statistical tests:

Parametric paired t-test (comparing mean availability): p-value = 0.0014.

Non-parametric Wilcoxon (paired) signed rank test (comparing median availability): p-value= 0.0039.

Conclusion: Since the p-value was found to be less than 0.05 in both tests, we do have enough statistical evidence ($\alpha = 0.05$) to reject the null. Therefore, the availability of originator brand version of OTC medicines is not same in chain and independent pharmacies.

Test S6:

Null hypothesis (4): Availability of generic versions of OTC medicines is same in chain and independent pharmacies.

Alternate hypothesis: Availability of generic versions of OTC medicines is not same in chain and independent pharmacies.

Alpha Significance level: 0.05

Statistical tests:

Parametric paired t-test (comparing mean availability): p-value = 0.0109.

Non-parametric Wilcoxon (paired) signed rank test (comparing median availability): p-value: 0.0039.

Conclusion: Since the p-value was found to be less than 0.05 in both tests, we do have enough statistical evidence ($\alpha = 0.05$) to reject the null. Therefore, the availability of generic versions of OTC medicines is not same in chain and independent pharmacies.

Test S7:

Null Hypothesis: Availability of originator brand and generic versions of prescription medicines is same in chain pharmacies.

Alternate hypothesis: Availability of originator brand and generic versions of prescription medicines is not same in chain pharmacies.

Alpha Significance level: 0.05

Statistical tests:

Parametric paired t-test (comparing mean availability): p-value = 0.0051.

Non-parametric Wilcoxon (paired) signed rank test (comparing median availability): p-value: 0.0020.

Conclusion: Since the p-value was found to be less than 0.05 in both tests, we do have enough statistical evidence (α = 0.05) to reject the null. Therefore, the availability of originator brand and generic versions of prescription medicines is not same in chain pharmacies.

Test S8:

Null Hypothesis: Availability of originator brand and generic versions of prescription medicines is same in independent pharmacies.

Alternate hypothesis: Availability of originator brand and generic versions of prescription medicines is not same in independent pharmacies.

Alpha Significance level: 0.05

Statistical tests:

Parametric paired t-test (comparing mean availability): p-value = 0.0020.

Non-parametric Wilcoxon (paired) signed rank test (comparing median availability): p-value: 0.0010.

Conclusion: Since the p-value was found to be less than 0.05 in both tests, we do have enough statistical evidence (α = 0.05) to reject the null. Therefore, the availability of originator brand and generic versions of prescription medicines is not same in independent pharmacies.

Test S9:

Null Hypothesis: Availability of originator brand version of prescription medicines is same in chain and independent pharmacies.

Alternate hypothesis: Availability of originator brand version of prescription medicines is not same in chain and independent pharmacies.

Alpha Significance level: 0.05

Statistical tests:

Parametric paired t-test (comparing mean availability): p-value = 0.0009.

Non-parametric Wilcoxon (paired) signed rank test (comparing median availability): p-value: 0.0034.

Conclusion: Since the p-value was found to be less than 0.05 in both tests, we do have enough statistical evidence (α = 0.05) to reject the null. Therefore, the availability of originator brand version of prescription medicines is not same in chain and independent pharmacies.

Test S10:

Null Hypothesis: Availability of generic version of prescription medicines is same in chain and independent pharmacies.

Alternate hypothesis: Availability of generic version of prescription medicines is not same in chain and independent pharmacies.

Alpha Significance level: 0.05

Statistical tests:

Parametric paired t-test (comparing mean availability): p-value = 1.00.

Non-parametric Wilcoxon (paired) signed rank test (comparing median availability): p-value: 1.00.

Conclusion: Since the p-value was found to be higher than 0.05 in both tests, we do have enough statistical evidence (α = 0.05) to reject the null. Therefore, the availability of generic version of prescription medicines is same in chain and independent pharmacies.

Results: Medicine Prices

Matched pair analysis

Test S11

Null Hypothesis: The distribution (median) of MPRs among originator (OB) and lowest price generic (LPG) version of OTC medicines is same.

Alternate hypothesis: The distribution (median) of MPRs among originator (OB) and lowest price generic (LPG) version of OTC medicines is not same.

Alpha Significance level: 0.05

Statistical tests:

Wilcoxon signed rank test (matched pair analysis): p-value = 0.0273.

Conclusion: Since the p-value was found to be less than 0.05, we do have enough statistical evidence (α = 0.05) to reject the null. Therefore, the distribution (Median) of MPRs of originator and lowest price generic version of OTC medicines is not same.

Test S12

Null Hypothesis: The distribution (median) of MPRs among OB and LPG versions of OTC medicines is same in independent pharmacies.

Alternate hypothesis: The distribution (median) of MPRs among OB and LPG versions of OTC medicines is not same in independent pharmacies.

Alpha Significance level: 0.05

Statistical tests:

Wilcoxon signed rank test (matched pair analysis): p-value = 0.0313.

Conclusion: Since the p-value was found to be less than 0.05, we do have enough statistical evidence (α = 0.05) to reject the null. Therefore, the distribution (median) of MPRs among OB and LPG versions of OTC medicines is not same in independent pharmacies.

Test S13

Null hypothesis: The distribution (median) of MPRs among OB and LPG versions of OTC medicines is same in chain pharmacies.

Alternate hypothesis: The distribution (median) of MPRs among OB and LPG versions of OTC medicines is not same in chain pharmacies.

Alpha Significance level: 0.05

Statistical tests:

Wilcoxon signed rank test (matched pair analysis): p-value = 0.0078.

Conclusion: Since the p-value was found to be less than 0.05, we do have enough statistical evidence (α = 0.05) to reject the null. Therefore, the distribution (median) of MPRs among OB and LPG versions of OTC medicines is not same in chain pharmacies.

Test S14

Null hypothesis: The distribution (median) of MPRs of OB versions of OTC medicines is same among chain and independent pharmacies.

Alternate hypothesis: The distribution (median) of MPRs of OB versions of OTC medicines is same among chain and independent pharmacies.

Alpha Significance level: 0.05

Statistical tests:

Wilcoxon signed rank test (matched pair analysis): p-value = 0.8125.

Conclusion: Since the p-value was found to be higher than 0.05, we do not have enough statistical evidence (α = 0.05) to reject the null. Therefore, the distribution (median) of MPRs of OB versions of OTC medicines is same among chain and independent pharmacies.

Test S15

Null hypothesis: The distribution (median) of MPRs of LPG versions of OTC medicines is same among chain and independent pharmacies.

Alternate hypothesis: The distribution (median) of MPRs of LPG versions of OTC medicines is not same among chain and independent pharmacies.

Alpha Significance level: 0.05

Statistical tests:

Wilcoxon signed rank test (matched pair analysis): p-value = 0.1953

Conclusion: Since the p-value was found to be higher than 0.05, we do not have enough statistical evidence (α = 0.05) to reject the null. Therefore, the distribution (median) of MPRs of LPG versions of OTC medicines is same among chain and independent pharmacies.

Test S16

Null hypothesis: The distribution (median) of MPRs of OB and LPG versions among prescription medicines are same.

Alternate hypothesis: The distribution (median) of MPRs among OB and LPG versions of prescription medicines are not same.

Alpha Significance level: 0.05

Statistical tests:

Wilcoxon signed rank test (matched pair analysis): p-value = 0.0273.

Conclusion: Since the p-value was found to be less than 0.05, we do have enough statistical evidence (α = 0.05) to reject the null. Therefore, the distribution (median) of MPRs among OB and LPG versions of prescription medicines are not same.

Test S17

Null hypothesis: The distribution (median) of MPRs among OB and LPG versions of prescription medicines are same in chain pharmacies.

Alternate hypothesis: The distribution (median) of MPRs among OB and LPG versions of prescription medicines are not same in chain pharmacies.

Alpha Significance level: 0.05

Statistical tests:

Wilcoxon signed rank test (matched pair analysis): p-value = 0.0313.

Conclusion: Since the p-value was found to be less than 0.05, we do have enough statistical evidence ($\alpha = 0.05$) to reject the null. Therefore, the distribution (median) of MPRs among OB and LPG versions of prescription medicines are not same in chain pharmacies.

Test S19

Null hypothesis: The distribution (median) of MPRs of LPG version of prescription medicines are same among chain and independent pharmacies.

Alternate hypothesis: The distribution (median) of MPRs of LPG version of prescription medicines are not same among chain and independent pharmacies.

Alpha Significance level: 0.05

Statistical tests:

Wilcoxon signed rank test (matched pair analysis): p-value = 0.3125.

Conclusion: Since the p-value was found to be higher than 0.05, we do not have enough statistical evidence ($\alpha = 0.05$) to reject the null. Therefore, the distribution (median) of MPRs of LPG version of prescription medicines are same among chain and independent pharmacies.

All medicines analysis (Two sample non-parametric test): OTC medicines

Test S20

Null hypothesis: The distribution (median) of MPRs among the OB and LPG versions of OTC medicines are same.

Alternate hypothesis: The distribution (median) of MPRs among the OB and LPG versions of OTC medicines are not same.

Alpha Significance level: 0.05

Statistical tests:

Wilcoxon rank sum test (two-sample test, unmatched): p-value = 0.0562.

Conclusion: Since the p-value was found to be higher than 0.05, we do not have enough statistical evidence (α = 0.05) to reject the null. Therefore, the distribution (median) of MPRs among the OB and LPG versions of OTC medicines are same. However the p-value (0.0562) was less than 0.06, there is borderline significance.

Test S21

Null hypothesis: The distribution (median) of MPR among the OB and LPG versions of OTC medicines is same in chain pharmacies.

Alternate hypothesis: The distribution (median) of MPR among the OB and LPG versions of OTC medicines is not same in chain pharmacies.

Alpha Significance level: 0.05

Statistical tests:

Wilcoxon rank sum test (two-sample test, unmatched): p-value = 0.1119.

Conclusion: Since the p-value was found to be higher than 0.05, we do not have enough statistical evidence (α = 0.05) to reject the null. Therefore, the distribution (median) of MPR among the OB and LPG versions of OTC medicines is same in chain pharmacies.

Test S22

Null hypothesis: The distribution (median) of MPR among the OB and LPG versions of OTC medicines is same in independent pharmacies.

Alternate hypothesis: The distribution (median) of MPR among the OB and LPG versions of OTC medicines is not same in independent pharmacies.

Alpha Significance level: 0.05

Statistical tests:

Wilcoxon rank sum test (two-sample test, unmatched): p-value = 0.0037.

Conclusion: Since the p-value was found to be less than 0.05, we do have enough statistical evidence (α = 0.05) to reject the null. Therefore, the distribution (median) of MPR among the OB and LPG versions of OTC medicines is not same in independent pharmacies.

Test S23

Null hypothesis: The distribution (median) of MPR of OB version of OTC medicines is same among chain and independent pharmacies.

Alternate hypothesis: The distribution (median) of MPR of OB version of OTC medicines is not same among chain and independent pharmacies.

Alpha Significance level: 0.05

Statistical tests:

Wilcoxon rank sum test (two-sample test, unmatched): p-value = 0.9626.

Conclusion: Since the p-value was found to be higher than 0.05, we do not have enough statistical evidence ($\alpha = 0.05$) to reject the null. Therefore, the distribution (median) of MPR of OB version of OTC medicines is not same among chain and independent pharmacies.

Test S24

Null hypothesis: The distribution (median) of MPR of LPG version of OTC medicines is same among chain and independent pharmacies.

Alternate hypothesis: The distribution (median) of MPR of LPG version of OTC medicines is not same among chain and independent pharmacies.

Alpha Significance level: 0.05

Statistical tests:

Wilcoxon rank sum test (two-sample test, unmatched): p-value = 0.5027.

Conclusion: Since the p-value was found to be higher than 0.05, we do not have enough statistical evidence ($\alpha = 0.05$) to reject the null. Therefore, distribution (median) of MPR of LPG version of OTC medicines is same among chain and independent pharmacies.

All medicines analysis (Two sample non-parametric test): Prescription medicines

Test S25

Null hypothesis: The distribution (median) of MPRs among OB and LPG version of prescription medicines is same.

Alternate hypothesis: The distribution (median) of MPRs among OB and LPG version of prescription medicines is not same.

Alpha Significance level: 0.05

Statistical tests:

Wilcoxon rank sum test (two-sample test, unmatched): p-value = 0.0422.

Conclusion: Since the p-value was found to be less than 0.05, we do have enough statistical evidence ($\alpha = 0.05$) to reject the null. Therefore, the distribution (median) of MPRs of OB and LPG version of prescription medicines is not same.

<u>Test S26</u>

Null hypothesis: The distribution (median) of MPRs among OB and LPG version of prescription medicines is same in chain pharmacies.

Alternate hypothesis: The distribution (median) of MPRs among OB and LPG version of prescription medicines is not same in chain pharmacies.

Alpha Significance level: 0.05

Statistical tests:

Wilcoxon rank sum test (two-sample test, unmatched): p-value = 0.0465.

Conclusion: Since the p-value was found to be less than 0.05, we do have enough statistical evidence ($\alpha = 0.05$) to reject the null. Therefore, the distribution (median) of MPRs among OB and LPG version of prescription medicines is not same in chain pharmacies.

<u>Test S27</u>

Null hypothesis: The distribution (median) of MPRs of OB version of prescription medicines is same among chain and independent pharmacies.

Alternate hypothesis: The distribution (median) of MPRs of OB version of prescription medicines is not same among chain and independent pharmacies.

Alpha Significance level: 0.05

Statistical tests:

Wilcoxon rank sum test (two-sample test, unmatched): p-value = 0.8636.

Conclusion: Since the p-value was found to be higher than 0.05, we do not have enough statistical evidence ($\alpha = 0.05$) to reject the null. Therefore, the distribution (median) of MPRs of OB version of prescription medicines is same among chain and independent pharmacies.

Test S28

Null hypothesis: The distribution (median) of MPRs of LPG version of prescription medicines is same among chain and independent pharmacies.

Alternate hypothesis: The distribution (median) of MPRs of LPG version of prescription medicines is not same among chain and independent pharmacies.

Alpha Significance level: 0.05

Statistical tests:

Wilcoxon rank sum test (two-sample test, unmatched): p-value = 0.2230.

Conclusion: Since the p-value was found to be higher than 0.05, we do not have enough statistical evidence ($\alpha = 0.05$) to reject the null. Therefore, the distribution (median) of MPRs of LPG version of prescription medicines is same among chain and independent pharmacies.

<u>List of price sources for the 2013 MSH Drug Price Indicator Guide</u>

Three primary types of suppliers and buyers are included in the Guide.

1. **Suppliers** who maintain a warehouse and supply items directly to customers. All of these suppliers provide a wider range of products than shown in this Guide.

2. **International development organizations (IDO)** that provide commodities to country programs these organizations and others support.

3. Buyers (usually public-sector agencies) that procure commodities through international competitive bidding, or tenders. These are actual prices obtained by the organizations listed and are included for comparison purposes.

**Source**	**Type**	**Details**
Action Medeor	Supplier	Prices are effective until publication of revised price list. Transport costs are charged individually according to expenses. Special packaging in cases costs Euro 26 per case. Minimum handling fee is Euro 50. Only serves non- profit organizations.
Action Medeor International Healthcare	Supplier	Action Medeor International Healthcare supplies the not- for-profit market (primarily in Tanzania) and does not supply to individuals or commercial pharmacies. A 3.5% service charge applies for all non- Tanzanian customers to cover bank charges and currency loss. No minimum quantities are required.
Amstelfarma	Supplier	Amstelfarma prices are given as an indication only. There are no minimum orders required or service charges.
Durbin PLC	Supplier	Durbin supplies pharmaceuticals and medical supplies to doctors, pharmacies, hospitals, pharmaceutical wholesalers and traders, military and government agencies, charities and other

		relief organizations worldwide. Minimum order UK £100.
IDA Foundation	Supplier	The IDA Foundation delivers high-quality essential medicines and medical supplies to low- and medium-income countries. As an independent and self-supporting foundation, IDA distributes more than 3,000 products to over 100 countries worldwide. Minimum order of US$5000. Orders under Euro 5,000 are charged a handling fee of 3%. Orders over Euro 5,000 are charged a handling fee of 1.5%. Prices are indicative and may change.
IMRES	Supplier	Minimum order quantity is US$1250.
Joint Medical Stores	Supplier	JMS supplies pharmaceuticals and medical supplies to mission health units and other NGOs in Uganda and does not export products. JMS operates on a not-for-profit basis, and price terms are cash-and-carry only. Prices are indicative.
Medical Export Group	Supplier	Prices are given as an indication only. There are no minimum orders required or service charges.
Medical Stores Department, Tanzania	Supplier	MSD sales prices are valid for all governmental health facilities, approved NGOs, mission hospitals, charitable hospitals and other health care providers in Tanzania, with approval from Ministry of Health & Social Welfare. Parastatal companies/organizations will pay 10% on top of these sales prices.

Mission for Essential Drugs and Supplies	Supplier	MEDS supplies drugs and medical supplies to nonprofit health care providers. Freight charges are added for deliveries outside of Kenya.
Missionpharma	Supplier	Prices are given as an indication only. There are no minimum orders required or service charges.
Global TB Drug Facility	IDO	Drugs are supplied through free grants, but are also available for purchase by governments and organizations wishing to take advantage of the quality, prices, and technical support offered through the GDF Direct Procurement Service. GDF uses procurement agents. Prices include the cost of the product as well as quality control costs and a GDF procurement agent fee. Costs for pre-shipment inspection, transport (air freight or sea freight) and insurance, are not included in the listed product prices.
Supply Chain Management System	IDO	SCMS is funded by the US government as part of the President's Emergency Plan for AIDS Relief (PEPFAR). The SCMS project can work anywhere in the world, but is initially focused on the 15 PEPFAR focus countries. In addition to serving any project or implementing partner supported by PEPFAR, SCMS can also supply projects supported by national governments and other donors, subject to individual arrangements.
UNICEF	IDO	As well as supporting UNICEF's ongoing programmatic activities, the UNICEF Supply function provides rapid supply response to emergencies. UNICEF also procures and supplies essential commodities on behalf of governments and other partners in their efforts. These procurement services can also include in-country logistics, capacity building, and project management. UNICEF prices are given as an indication only.

UN Population Fund	IDO	UNFPA conducts annual tenders to purchase goods for their field offices and on behalf of external clients in more than 168 countries, mainly in the developing world. UNFPA serves only governments and nonprofit organizations. UNFPA charges a 5% administration fee and a 0.132% insurance fee to non-UNFPA buyers. There is a minimum order requirement of US$5,000 per item.
U.S. Agency for International Development Central Contraceptive Procurement Project	IDO	The USAID | CCP Project, established in 1990, serves as the central procurement mechanism for USAID Missions to purchase high quality contraceptives, condoms, and other essential public health supplies. The USAID | CCP Project (Central Contraceptive Procurement) also administers the Commodity Fund, which serves to increase condom availability and use by making condoms for HIV prevention free of charge to nonfocus programs. Since 2006, CCP also has provided other essential public health pharmaceuticals, diagnostics, and medical equipment at the request of USAID Missions. A handling fee is applied to each order and this is based on the type of commodity procured.
Barbados Drug Service	Buyer	The Barbados Drug Service conducts an annual, domestic, open tender for pharmaceuticals and medical supplies.
Ministerio de Salud y Deportes del Bolivia	Buyer	The Bolivia Ministry of Health and Sports conducts an annual, open, international tender for pharmaceuticals and medical supplies.
Botswana Public Procurement and Asset Disposal Board	Buyer	The Public Procurement and Asset Disposal Board of Botswana conducts open and closed domestic and international tenders on a regular basis.

Caja Costarricense de Seguro Social	Buyer	Costa Rica Social Security conducts an annual, open, international tender and negotiates contracts for drugs and medical supplies for its own facilities.
Clinton Health Access Initiative	Buyer	The Clinton Health Access Initiative contributed price information from several partners who are working together to scale up treatment for diarrhea.
Democratic Republic of Congo Integrated Health Program (IHP)	Buyer	The Democratic Republic of Congo Integrated Health Project (IHP) conducts an annual, closed, international tender for pharmaceuticals and medical supplies.
República Dominicana Central de Apoyo Logístico Programa de Medicamentos Esenciales (PROMESE/CAL)	Buyer	The Dominican Republic Essential Medicine Program Central Logistics Support conducts an annual open, domestic tender for pharmaceuticals and medical supplies. =
Mission for Essential Medical Supplies (MEMS), Tanzania	Buyer	MEMS awards an annual drug and medical supply contract to a prime vendor, after an open domestic pre-qualification process and a closed tender process.
Namibia Central Medical Stores	Buyer	The Namibia Central Medical Stores conducts an open, international tender every two years for pharmaceuticals and medical supplies and once a year for anti-retrovirals.

Organisation of Eastern Caribbean States Pharmaceutical Procurement Service (OECS/PPS)	Buyer	The OECS/PPS provides its services to member countries of the Organisation of Eastern Caribbean States only. For these nine countries, OECS/PPS negotiates contracts and purchases through a closed, international tender every 18 months.
Perú Ministerio de Salud	Buyer	The Peru Ministry of Health (MINSA) conducts an annual, domestic, open tender for pharmaceuticals and medical supplies.
El Sistema de Integración Centroamericana (SICA)	Buyer	The System of Central American Integration (SICA) conducts an annual open, international tender for selected pharmaceuticals.
South Africa Department of Health	Buyer	The South Africa Department of Health conducts two-year domestic, open tenders on a rolling basis for pharmaceuticals and medical supplies.
Sudan National Health Insurance Fund	Buyer	The National Health Insurance Fund of Sudan conducts annual, open, domestic tenders for pharmaceuticals and medical supplies.

Reference:

1. United States Office of Management and Budget. OMB Bulletin No. 13-01. Washington D.C., 2013.
